# Impact of T-tube placement on hepaticojejunostomy stenosis after pancreatic surgery

**DOI:** 10.1186/s12893-026-03763-w

**Published:** 2026-04-22

**Authors:** Jennifer Herzog-Niescery, Willy Chedjou, Ilgar Aghalarov, Waldemar Uhl, Torsten Herzog

**Affiliations:** 1 Department of Anesthesiology, St. Josef Hospital, Ruhr University Bochum, Bochum, Germany; 2 Department of General and Visceral Surgery, St. Josef Hospital, Ruhr University Bochum, Bochum, Germany; 3 Department of Anesthesiology, Intensive Care and Pain Therapy, University Hospital Knappschaftskrankenhaus, Bochum, Germany; 4 Department of General and Visceral Surgery, Knappschaft-Kliniken, Recklinghausen, Germany

**Keywords:** Hepaticojejunostomy, Pancreatic surgery, Pancreaticoduodenectomy, T-tube drainage, Biliary stricture, Anastomotic stenosis, Bile duct diameter, Postoperative complications

## Abstract

**Background:**

Hepaticojejunostomy stenosis represents a clinically relevant long-term complication after pancreatic surgery and may result in recurrent cholangitis and impaired quality of life. T-tube drainage is occasionally used during biliary reconstruction, particularly in technically challenging anastomoses involving narrow bile ducts; however, its impact on the development of hepaticojejunostomy stenosis remains controversial. This study aimed to evaluate the association between intraoperative T-tube placement and hepaticojejunostomy stenosis after pancreatic surgery.

**Methods:**

This retrospective single-center cohort study included patients who underwent pancreatic surgery with hepaticojejunostomy between 2016 and 2018 at a high-volume pancreatic surgery center. Patients were stratified according to intraoperative T-tube placement. The primary endpoint was the incidence of hepaticojejunostomy stenosis, defined as radiologically confirmed biliary obstruction at the anastomotic site combined with persistent elevation of cholestatic laboratory parameters. Secondary endpoints included postoperative complications classified according to the Clavien–Dindo system and longitudinal cholestasis parameters during follow-up.

**Results:**

A total of 142 patients were included, of whom 79 (55.6%) underwent hepaticojejunostomy with T-tube placement. Narrow bile ducts (< 5 mm) were significantly more common in the T-tube group (78.5% vs. 9.5%, *p* < 0.001). The incidence of hepaticojejunostomy stenosis did not differ significantly between patients with and without T-tube placement (6.4% vs. 13.5%, *p* = 0.475). T-tube placement was associated with a higher incidence of early postoperative major complications, whereas rates of biliary anastomotic leakage were comparable between groups. Longitudinal analysis of cholestatic laboratory parameters revealed no significant differences during follow-up.

**Conclusions:**

T-tube placement during hepaticojejunostomy in pancreatic surgery was not associated with an increased risk of hepaticojejunostomy stenosis, despite its predominant use in patients with narrow bile ducts. These findings suggest that selective T-tube use does not adversely affect long-term biliary patency.

## Introduction

Pancreatic head resection is the treatment of choice for periampullary malignant tumors, including pancreatic ductal adenocarcinoma, distal cholangiocarcinoma, ampullary carcinoma, and duodenal cancer [1]. Despite substantial improvements in surgical technique and perioperative management, pancreatic surgery still is associated with high postoperative morbidity and mortality [2, 3]. While early complications such as postoperative pancreatic fistula and delayed gastric emptying are well described [4, 5], late biliary complications remain comparatively underreported.

Reconstruction after pancreaticoduodenectomy typically involves three anastomoses: pancreas to small bowel or stomach (pancreaticojejunostomy or pancreaticogastrostomy, respectively), bile duct to jejunum (hepaticojejunostomy (HJ)), and stomach to jejunum (gastrojejunostomy). Although leakage of the biliary anastomosis represents an early postoperative complication, stenosis of the HJ constitutes a clinically relevant long-term complication [6]. HJ stenosis may result in recurrent cholangitis, secondary biliary cirrhosis, and significant impairment of quality of life, often necessitating repeated interventional or surgical treatments [7, 8]. Reported incidences of HJ stenosis after pancreaticoduodenectomy range from approximately 2% to 5%, with diagnosis typically occurring more than one year postoperatively [6, 9, 10].

The pathogenesis of HJ stenosis is considered multifactorial and includes ischemic injury, technical aspects of anastomotic construction, inflammatory processes, and bile duct diameter [11, 12]. Especially the reconstruction of a narrow and thin-walled bile duct is technically demanding and has been identified as a major risk factor for subsequent anastomotic stricture formation. Several studies reported that a bile duct diameter < 5 mm significantly increases the risk of biliary complications after HJ reconstruction [9, 10, 13, 14].

Intraoperative biliary drainage has been established to facilitate biliary reconstruction in technically difficult anastomosis, most commonly by placing a T-tube across the HJ. Benefits include biliary decompression, postoperative cholangiography, and controlled external drainage in case of an anastomotic leak, potentially avoiding early re-surgery [15–17]. However, the use of T-tubes remains controversial, particularly regarding long-term outcomes. Evidence from hepatobiliary and transplant surgery suggests both beneficial and adverse effects, with conflicting results concerning early complications and late anastomotic strictures [18–22]. Importantly, most available evidence originates from liver transplantation or hepatic resections, whereas data focusing specifically on pancreatic surgery are scarce. Given the limited long-term survival of patients after pancreaticoduodenectomy, late biliary complications may be underrecognized, and systematic evaluations of HJ stenosis as a long-term outcome are lacking [6, 9]. Accordingly, current guidelines provide no clear recommendations regarding the use of T-tube drainage during HJ in pancreatic surgery.

Against this background, this study aimed to assess the association between intraoperative T-tube placement during HJ and biliary complications after pancreatic surgery. In a retrospective cohort from a high-volume center, patients with and without T-tube drainage were compared. We hypothesized that T-tube placement is not associated with an increased incidence of HJ stenosis during long-term follow-up.

## Methods

This retrospective single-center cohort study was conducted between 2016 and 2018 at a German high-volume pancreatic surgery center. The institution performs approximately 350 pancreatic procedures annually. The study was approved by the institutional review board of the Ruhr University Bochum (approval no. 21-7362-RR) and performed in accordance with the latest version of the Declaration of Helsinki. All patients provided written informed consent for surgery and data collection.

### Inclusion and exclusion criteria

All patients who underwent pancreatic surgery with construction of a HJ during the study period were screened for eligibility. Patients were included if complete preoperative and postoperative data were available, including documentation of bile duct diameter (< 5 mm or ≥ 5 mm), and if follow-up of at least twelve months was documented. Patients with insufficient follow-up, major documentation gaps, or alternative biliary reconstruction techniques were excluded.

### Data collection

Data were extracted from electronic medical records and operative reports. Collected variables included age, sex, relevant comorbidities, surgical indication, type of surgical procedure, bile duct diameter, postoperative complications, T-tube placement and timing of removal, laboratory cholestasis parameters, and radiological findings.

Eligible patients were divided into two groups according to intraoperative biliary drainage: patients with T-tube placement (T-tube group) and patients without T-tube placement (no T-tube group).

### Surgical procedures and biliary reconstruction

Surgical procedures included standard pancreaticoduodenectomy (Whipple procedure), pylorus-preserving pancreaticoduodenectomy (PPPD), total pancreatectomy, and non-resective procedures such as biliary and gastroenteric bypass.

HJ was constructed using a standardized end-to-side, single-layer anastomotic technique in all patients. Interrupted monofilament sutures (PDS 5 − 0) were routinely used for the anastomosis. In cases with a very small bile duct, PDS 6 − 0 sutures were used at the discretion of the surgeon to allow more delicate approximation of the duct wall.

**Fig. 1:**
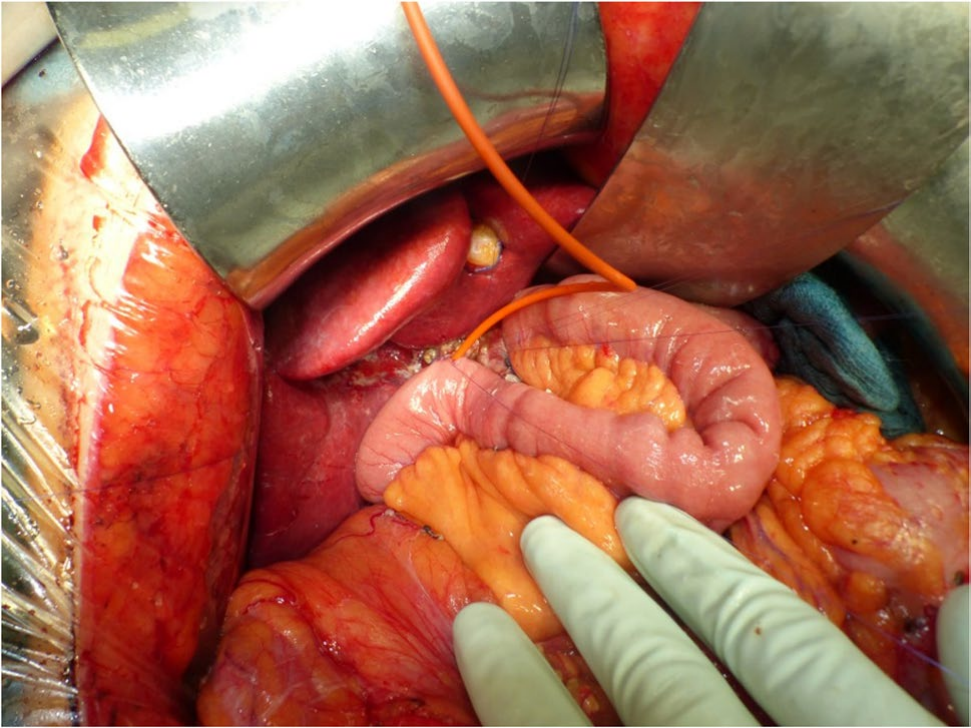
T-tube placement in bile duct and jejunal lumen

Following enterotomy of the jejunal loop, the bile duct was suspended using lateral stay sutures to optimize visualization and alignment. Independent of the placement of a T-tube, the posterior wall of the anastomosis was fashioned first. Seven interrupted sutures were placed sequentially and subsequently tied to complete the posterior layer. Thereafter, seven interrupted sutures were preplaced along the anterior wall. In cases without T-tube placement, the anterior wall sutures were tied directly, thereby completing the anastomosis. In cases with T-tube placement, the T-tube was inserted transanastomotically after preplacement of the anterior wall sutures and before knotting. One horizontal limb was positioned within the bile duct and the other within the jejunal lumen of the HJ. The vertical limb of the T-tube was then exteriorized transcutaneously through the right upper abdomen. Following correct positioning of the T-tube, the anterior wall sutures were tied (Fig. 1).

T-tube placement was performed selectively based on intraoperative anatomical and technical considerations. A narrow bile duct with a diameter of less than 5 mm - particularly in the presence of a thin or fragile duct wall requiring fine suture material (e.g., 6 − 0 polydioxanone) - represented the primary indication for T-tube insertion. However, the final decision was made at the surgeon’s discretion, taking into account overall intraoperative conditions and perceived anastomotic difficulty, and was not based on the underlying diagnosis [15, 23].

Postoperative imaging to assess T-tube position and biliary integrity was performed between postoperative days 5 and 7. In the absence of bile leakage, the T-tube was subsequently clamped for six to eight weeks. Removal was performed during a planned inpatient stay under antibiotic prophylaxis after confirmation of an uncomplicated course.

### Postoperative complications

Postoperative complications were classified according to the modified Clavien-Dindo classification [24]. Minor complications were defined as grade I–II, including events that require not more than standard supportive measures, parenteral nutrition, or pharmacological treatment. Major complications were defined as grade III–V events. Grade III complications required surgical, radiological, or endoscopic intervention. Postoperative pancreatic fistulas and biliary fistulas were classified according to their clinical severity and required management. Only complications requiring interventional, endoscopic, or surgical treatment were considered major complications, whereas biochemical leaks and conservatively managed fistulas were classified as minor complications. Grade IV complications comprised life-threatening events requiring intensive care management, while grade V was defined as death.

### Hepaticojejunostomy stenosis

HJ stenosis was defined as radiologically via computed tomography and /or magnetic resonance cholangiopancreatography confirmed obstruction at the level of the HJ, accompanied by intrahepatic cholestasis and a persistent elevation of cholestatic laboratory parameters to at least twice the upper limit of normal. These included gamma-glutamyl transferase, alkaline phosphatase, and total bilirubin.

### Follow-up interval

Patients were routinely followed up at 6, 12, and 24 months after surgery. At each follow-up visit, clinical status and cholestatic laboratory parameters were evaluated. Radiological imaging was performed if clinically indicated. HJ stenosis was diagnosed based on clinical findings, cholestatic laboratory parameters, and radiological evidence of biliary obstruction. Whenever available, a minimum postoperative follow-up of 24 months was considered.

### Statistical analysis

Descriptive statistics were used to characterize the study cohort. Continuous variables are reported as means with standard deviations or medians with interquartile ranges, depending on data distribution. Categorical variables were compared using the chi-square test or Fisher’s exact test, as appropriate. Continuous variables were analyzed using Student’s t-test or the Mann–Whitney U test. A p-value < 0.05 was considered statistically significant.

To evaluate the independent association between T-tube placement and HJ stenosis, univariate and multivariate logistic regression analyses were performed. The binary outcome variable was defined as radiologically confirmed HJ stenosis (CT and/or MRCP) at any follow-up timepoint (3, 6, 12, or 24 months). In the univariate analysis, the following covariates were assessed: T-tube placement, age (continuous, per year), sex, bile duct diameter (< 5 mm vs. ≥5 mm), and comorbidity status. For the multivariate model, covariates were selected a priori based on the following criteria: T-tube placement was included as the primary exposure of interest; narrow bile duct diameter was included as a key clinical confounder given its known association with both T-tube use and anastomotic stricture risk; and age was included based on borderline significance in univariate analysis (*p* < 0.10). The number of covariates was constrained to three to maintain an adequate events-per-variable (EPV) ratio (27 events / 3 variables = 9 EPV). Results are reported as odds ratios (OR) and adjusted odds ratios (aOR) with 95% confidence intervals (CI).

A post-hoc power analysis was performed to assess the statistical power of the available sample size. Statistical analyses were conducted using SPSS Statistics for macOS (version 29.0; IBM Corp., Armonk, NY, USA).

## Results

In the study period 617 patients underwent pancreatic surgery. Based on in- and exclusion criteria, 142 patients were included in the final analysis. Of these, 79 patients (55.6%) underwent HJ with intraoperative T-tube placement, while 63 patients (44.4%) underwent HJ without T-tube placement.

### Patient characteristics

Overall, 38.7% of the patients were female and 61.3% were male. Sex distribution did not differ significantly between groups (*p* = 0.75). The mean age at surgery was 63.6 ± 12.6 years (range 23–86 years), with comparable age distributions between the two groups. Underlying diseases are summarized in Table 1. Malignant disease was the indication for surgery in approximately 80% of patients. Pancreatic head adenocarcinoma was the most common malignant diagnosis, followed by ampullary carcinoma. Chronic pancreatitis was the most frequent benign indication. The distribution of malignant versus benign disease differed significantly between groups; it was 69.6% in group A and 90.5% in group B (*p* = 0.006). Overall, pylorus-preserving pancreaticoduodenectomy was most frequently performed, followed by pancreatectomy, and double bypass procedures. The distribution of surgical interventions within the two groups is shown in Table 2.

**Table 1:** Underlying diagnoses of the study cohort stratified by T-tube placement. The distribution of benign and malignant pancreatic diseases differed significantly between patients with T-tube and those without T-tube placement. Malignant diagnoses were more frequent in patients without T-tube, whereas benign diseases were more commonly observed in patients with T-tube. IPMN = Intraductal papillary mucinous neoplasms; SPN = Solid pseudopapillary neoplasm of the pancreas

Underlying disease	All *n* = 142	with T-tube *n* = 79	without T-tube *n* = 63	*p*-value
Benign diseases	20.4% (29/142)	29.1% (23/79)	9.5% (6/63)	0.006
Chronic pancreatitis	12% (17/142)	16.5% (13/79)	6.3% (4/63)	
IPMN tumors	6.3% (9/142)	8.9% (7/79)	3.2% (2/63)	
Serous cystadenoma	1.4% (2/142)	2.5% (2/79)	0%	
Ampullary adenoma	0.7% (1/142)	1.3% (1/79)	0%	
Malignant tumors	78.9% (112/142)	69.6% (55/79)	90.5% (57/63)	0.006
Pancreatic head carcinoma	61.3% (87/142)	51.9% (41/79)	73% (46/63)	
Ampullary carcinoma	12.7% (18/142)	8.9% (7/79)	17.5% (11/63)	
Neuroendocrine tumors	3.5% (5/142)	6.3% (5/79)	0%	
Duodenal carcinoma	0.7% (1/142)	1.3% (1/79)	0%	
SPN of the pancreas	0.7% (1/142)	1.3% (1/79)	0%	

**Table 2:** Surgical procedures by study group (percentages and absolute numbers). PPPD = Pylorus-preserving pancreaticoduodenectomy

Surgical procedure	All *n* = 142	with T-tube *n* = 79	without T-tube *n* = 63	*p*-value
PPPD	52.1% (74/142)	51.9% (41/79)	52,4% (33/63)	n. s.
Pancreatectomy	23.2% (33/142)	32.9% (26/79)	11.1% (7/63)	0.002
Double bypass procedure	16.9% (24/142)	7.6% (6/79)	28.6% (18/63)	0.001
Standard pancreaticoduodenectomy	7% (10/142)	6.3% (5/79)	7.9% (5/63)	n. s.
Bilioenteric anastomosis	0.7% (1/142)	1.3% (1/79)	0%	n. s.

Preoperative characteristics potentially associated with biliary reconstruction were further analyzed. A narrow bile duct (< 5 mm) was significantly more common in the T-tube group than in the non–T-tube group (78.5% vs. 9.5%; *p* = 0.001). Chronic pancreatitis as the underlying disease was present in 16.5% of patients in the T-tube group and in 6.3% of patients in the no T-tube group. An episode of acute pancreatitis within three months prior to surgery was rare and occurred in three patients in the T-tube group and in two patients in the no T-tube group (*p* = 1.00). Preoperative cholangitis was documented in two patients in the T-tube group and in six patients in the no T-tube group, without a statistically significant difference between groups (*p* = 0.14). A preoperative endoscopic biliary stent was present in 12.7% of patients in the T-tube group and in 25.4% of patients in the no T-tube group, without a statistically significant difference between groups (*p* = 0.051).

### Postoperative complications

Major postoperative complications occurred in 9.9% of all patients and were significantly more frequent in patients with than without T-tube (*p* < 0.001). However, the incidence of biliary anastomotic insufficiency did not differ significantly between groups (2.8% with T-tube vs. 0% without T-tube, *p* = 0.129). Minor complications occurred in approximately 47%, most commonly delayed gastric emptying, and did not differ between the groups (Table 3).

**Table 3:** Postoperative complications stratified by severity and by T-tube. Values are presented as percentages and absolute numbers. POPF = Postoperative pancreatic fistula

Postoperative complications	All *n* = 142	with T-tube *n* = 79	without T-tube *n* = 63	*p*-value
Major complications	9.9% (14/142)	16.5% (13/79)	1.6% (1/63)	< 0.001
Bilioenteric anastomotic leakage	2.8% (4/142)	5.1% (4/79)	0%	0.129
Respiratory failure	1.4% (2/142)	2.5% (2/79)	0%	0.503
Bleeding requiring blood transfusion	1.4% (2/142)	2.5% (2/79)	0%	0.503
Infected subhepatic hematoma	0.7% (1/142)	1.3% (1/79)	0%	1
Erosive bleeding left hepatic artery	0.7% (1/142)	1.3% (1/79)	0%	1
Hepatic ischemia	0.7% (1/142)	1.3% (1/79)	0%	1
Gastric perforation	0.7% (1/142)	1.3% (1/79)	0%	1
Anastomotic ulcer	0.7% (1/142)	1.3% (1/79)	0%	1
Necrotizing pancreatitis	0.7% (1/142)	0%	1.6% (1/63)	1
Minor complications	46.8% (66/142)	46.8% (37/79)	46.0% (29/63)	0.920
Postoperative gastrointestinal atony	13.4% (19/142)	11.4% (9/79)	15.9.% (10/63)	0.466
POPF grade A	9.2% (13/142)	7.6% (6/79)	11.1% (7/63)	0.562
POPF grade B	4.9% (7/142)	7.6% (6/79)	1.6% (1/63)	0.132
Wound healing disorders	4.9% (7/142)	3.8% (3/79)	6.3% (4/63)	0.670
Bile leak grade A	2.8% (4/142)	1.3% (1/79)	4.8% (3/63)	0.322
Bile leak grade B	2.8% (4/142)	5.1% (4/79)	0%	0.129
Cholangitis	2.1% (3/142)	2.5% (2/79)	1.6% (1/63)	1
Pneumonia	1.4% (2/142)	2.5% (2/79)	0%	0.503
Lymphatic fistula	1.4% (2/142)	1.3% (2/79)	0%	0.503
Postoperative bleeding	2.1% (3/142)	1.3% (2/79)	1.6% (1/63)	1
Chylous fistula	1.4% (2/142)	0%	3.2% (2/63)	0.195

### Cholestasis parameters

Preoperatively and postoperatively, cholestasis parameters (GGT, AP, bilirubin) were significantly higher in patients without T-tube compared with T-tube placement (*p* = 0.003, *p* < 0.001, and *p* = 0.005, respectively). This likely reflects the higher proportion of patients with preoperative biliary obstruction and dilated bile ducts in the no T-tube group. During follow-up at 3, 6, 12, and 24 months, mean and median values showed considerable variability with several outliers in both groups. Despite a tendency toward higher values in the no T-tube group, no statistically significant differences were observed at any follow-up interval.

### T-tube removal

T-tube removal was performed as scheduled after six weeks in 53.2% of patients. Delayed removal occurred in 44.3%, mainly due to postoperative complications (Table 3). Early removal was required in 2.5% of patients. No significant association was found between timing of T-tube removal and the development of HJ stenosis during follow-up (all *p* > 0.196).

### Radiological assessment and HJ

Radiological follow-up (CT and/or MRCP) was available for 59.9 (with T-tube) and 71.8% (without T-tube) of patients across follow-up intervals. The incidence of radiologically confirmed HJ stenosis was 6.4% with T-tube and 13.5% without T-tube and did not differ significantly between groups at 3, 6, 12, or 24 months (all *p* > 0.35). In patients with malignant primary disease, available follow-up imaging was reviewed to exclude tumor recurrence as the cause of biliary obstruction.

In univariate logistic regression analysis, T-tube placement was not significantly associated with HJ stenosis (OR 0.57, 95% CI 0.25–1.33, *p* = 0.197). Among the covariates analyzed, age showed a borderline association (OR 1.04, 95% CI 1.00–1.09, *p* = 0.051), as did narrow bile duct diameter (OR 0.47, 95% CI 0.20–1.14, *p* = 0.097). In multivariate analysis adjusting for bile duct diameter and age, T-tube placement remained non-significant (adjusted OR 0.87, 95% CI 0.27–2.84, *p* = 0.817).

Most HJ stenoses were transient and associated with mild cholestasis, responding well to conservative treatment. Higher-grade HJ stenoses were mainly related to tumor recurrence or metastatic disease rather than technical failure of the anastomosis.

## Discussion

The present retrospective cohort study investigated the association between intraoperative T-tube placement during pancreatic surgery and the occurrence of HJ stenosis. In this high-volume single-center cohort, T-tube placement was not associated with an increased incidence of HJ stenosis during long-term follow-up. However, a higher rate of early postoperative complications was observed in patients receiving T-tube placement.

Postoperative HJ stenosis is a clinically relevant long-term complication after pancreatic surgery, frequently associated with recurrent cholangitis, impaired quality of life, and the need for endoscopic or surgical interventions. Its pathogenesis is considered multifactorial, however, besides preoperative biliary drainage, postoperative bile leakage, and complex biliary anatomy, the surgical technique may be a risk factor [6, 25–27]. Within this context, the role of T-tube drainage remains controversial. Although T-tube placement has traditionally been advocated in technically difficult biliary anastomoses to decompress the biliary system, concerns have been raised regarding chronic inflammation, scarring along the drainage tract, and late anastomotic stenosis associated with T-tubes [9, 16].

From a surgical perspective, the rationale for T-tube placement is not necessarily the prevention of bile leakage itself but rather the mitigation of its clinical consequences in technically demanding anastomoses. In difficult HJ, particularly in the presence of small or fragile bile ducts, T-tube drainage may provide temporary decompression of the biliary system and facilitate controlled external drainage in the event of an early leak. Previous studies showed that although T-tube placement did not significantly reduce the incidence of bile leakage, it was associated with a lower rate of reoperation due to reduced severity of bile leaks in complex anastomoses [28]. These findings support the concept that T-tubes may serve as a safety measure in technically challenging reconstructions rather than as a strategy to prevent biliary complications per se.

In our cohort, T-tube placement was predominantly used in patients with narrow bile ducts. According to previous studies, such patients would be expected to have a higher risk of developing HJ stenosis [9, 10], but we observed no increased HJ stenosis rate. On the contrary, the numerically higher stenosis rate in the non–T-tube group suggest that T-tube placement does not appear to exacerbate the risk of late anastomotic stenosis in pancreatic surgery. Moreover, it suggests that bile duct diameter alone may not be sufficient to predict late anastomotic failure. These findings challenge the assumption that T-tube placement inherently promotes fibrotic remodeling or scarring of the biliary anastomosis.

The distribution of underlying diagnoses differed significantly between groups. Patients in the T-tube group had a significantly higher proportion of benign disease, particularly chronic pancreatitis, which is known to be associated with narrow bile ducts [15]. While some studies have identified benign disease as a risk factor for biliary complications [10], others found no association between tumor malignancy and HJ stenosis [6]. In the present analysis, differences in underlying pathology did not translate into a higher stenosis rate, suggesting that disease etiology alone may not be a decisive determinant of long-term biliary outcome.

With respect to postoperative morbidity, we observed a higher incidence of early postoperative complications in patients with T-tube drainage, including bilioenteric anastomotic leakage and clinically relevant postoperative pancreatic fistula. These findings are in line with previous reports suggesting an association between T-tube use and increased rates of bile leakage or biliary complications [29]. Moreover, Javed et al. demonstrated that postoperative bile leakage and pancreatic fistula were significantly more common in patients who subsequently developed biliary stenoses [27]. While all pancreatic fistulas in the present cohort were managed conservatively, their occurrence may still contribute to local inflammation and impaired anastomotic healing.

A subgroup analysis addressed the timing of T-tube removal, as it is a critical period associated with increased biliary complications [30, 31]. However, no robust evidence was observed, though sample size was small. Prospective studies specifically designed to evaluate optimal drainage duration are warranted.

Laboratory follow-up demonstrated a trend toward normalization of cholestatic parameters after T-tube removal, could indicate that prolonged indwelling drainage may contribute to biliary inflammation or functional obstruction. These controversial findings are in line with results from hepatobiliary and transplant surgery. Meta-analyses and large registry studies have reported comparable or even higher rates of HJ stenoses in patients with T-tubes compared to those patients without T-tube [20, 32]. Others suggest a benefit from selective use in high-risk anastomoses [33]. Alternative approaches, such as internal or internal–external stenting (e.g., Neuhaus drainage), have been proposed for complex biliary reconstructions, although robust evidence supporting their routine use is still lacking [26, 34]. This dual role of T-tubes, potentially protective in the early postoperative phase but pro-inflammatory in the longer term, may help explain the conflicting results reported in the literature.

### Limitations

This study has several limitations. First, the retrospective design inherently limits control of confounding and allows only the assessment of associations rather than causal relationships.

Second, the small sample size and the low incidence of HJ stenosis limit the statistical power (post-hoc power ~ 30%), and clinically relevant differences between groups may therefore have remained undetected.

Third, this was a single-center study conducted at a high-volume pancreatic surgery center. While this ensures a high level of surgical expertise, the findings may not be fully generalizable to centers with different case volumes or surgical practices.

Fourth, long-term follow-up in patients undergoing pancreatic surgery is challenging due to limited overall survival, particularly in malignant disease. Late biliary complications such as HJ stenosis may therefore be underreported, potentially leading to an underestimation of their true incidence.

Finaly, metastases of hepatoduodenal ligament lymph nodes were not systematically documented in the operative reports and therefore could not be included in the analysis. Although radiological follow-up did not reveal evidence of local tumor recurrence causing biliary obstruction, the possibility of malignant influence on biliary narrowing cannot be completely excluded due to the retrospective study design.

Despite these limitations, this study provides real-world data from a high-risk cohort with a high proportion of narrow bile ducts and may serve as a foundation for future prospective, ideally multicenter studies evaluating the role of T-tube drainage in HJ.

## Conclusion

In this retrospective cohort study, T-tube placement during HJ in pancreatic surgery was not associated with an increased incidence of HJ stenosis or long-term cholestasis, even in patients with narrow bile ducts. Although T-tubes were primarily used in technically challenging anastomoses, their use did not result in higher rates of late biliary stenoses. The increased rate of early postoperative complications underscores the need for cautious application. Further prospective, adequately powered multicenter studies are required to clarify the role of T-tube drainage in this setting.

## Data Availability

De-identified individual participant data and complete statistical analysis code that support the findings of this study are available from the corresponding author upon request.

## Abbreviations

HJ: Hepaticojejunostomy
PD: Pancreaticoduodenectomy
PPPD: Pylorus-preserving pancreaticoduodenectomy
CT: Computed tomography
MRCP: Magnetic resonance cholangiopancreatography
POPF: Postoperative pancreatic fistula
EPV: Events-per-variable
OR: Odds ratios
aOR: Adjusted odds ratios
CI: Confidence intervals

## References

[1] Hank T, Klaiber U, Sahora K, Schindl M, Strobel O. Surgery for periampullary pancreatic cancer. Chirurg. 2021;92(9):776–87. 10.1007/s00104-021-01462-1. 34259884 10.1007/s00104-021-01462-1 PMC8384803

[2] Cameron JL, He J. Two thousand consecutive pancreaticoduodenectomies. J Am Coll Surg. 2015;220(4):530–6. 10.1016/j.jamcollsurg.2014.12.031. 25724606 10.1016/j.jamcollsurg.2014.12.031

[3] Mischinger HJ, Werkgartner G, Kornprat P, Marsoner K, Wagner D, Cerwenka H, et al. Complications in pancreatic surgery. Wien Klin Mag. 2018;21(3):98–107. 10.1007/s00740-018-0226-1.

[4] Bassi C, Dervenis C, Butturini G, Fingerhut A, Yeo C, Izbicki J, et al. Postoperative pancreatic fistula: an international study group (ISGPF) definition. Surgery. 2005;138(1):8–13. 10.1016/j.surg.2005.05.001. 16003309 10.1016/j.surg.2005.05.001

[5] Wente MN, Bassi C, Dervenis C, Fingerhut A, Gouma DJ, Izbicki JR, et al. Delayed gastric emptying (DGE) after pancreatic surgery: a suggested definition by the International Study Group of Pancreatic Surgery (ISGPS). Surgery. 2007;142(5):761–8. 10.1016/j.surg.2007.05.005. 17981197 10.1016/j.surg.2007.05.005

[6] House MG, Cameron JL, Schulick RD, Campbell KA, Sauter PK, Coleman J, et al. Incidence and outcome of biliary strictures after pancreaticoduodenectomy. Ann Surg. 2006;243(5):571–6. 10.1097/01.sla.0000216285.07069.fc. 16632990 10.1097/01.sla.0000216285.07069.fc PMC1570556

[7] Keim V, Klar E, Poll M, Schoenberg MH. The pancreatic surgery patient. Dtsch Arztebl Int. 2009;106(48):789–94. 10.3238/arztebl.2009.0789. 20038981 10.3238/arztebl.2009.0789 PMC2797397

[8] Park JW, Jang JY, Kim EJ, Kang MJ, Kwon W, Chang YR, et al. Effects of pancreatectomy on nutritional state, pancreatic function and quality of life. Br J Surg. 2013;100(8):1064–70. 10.1002/bjs.9146. 23616030 10.1002/bjs.9146

[9] Duconseil P, Turrini O, Ewald J, Berdah SV, Moutardier V, Delpero JR. Biliary complications after pancreaticoduodenectomy: skinny bile ducts are surgeons’ enemies. World J Surg. 2014;38(11):2946–51. 10.1007/s00268-014-2698-5. 25011578 10.1007/s00268-014-2698-5

[10] Malgras B, Duron S, Gaujoux S, Dokmak S, Aussilhou B, Rebours V, et al. Early biliary complications following pancreaticoduodenectomy: prevalence and risk factors. HPB (Oxford). 2016;18(4):367–74. 10.1016/j.hpb.2015.10.012. 27037207 10.1016/j.hpb.2015.10.012 PMC4814603

[11] Zöpf T, Gerken G. Biliary strictures after liver transplantation. Gastroenterologe. 2008;3(1):39–44. 10.1007/s11377-007-0131-4.

[12] Heidenhain C, Rösch R, Neumann UP. Hepatobiliary anastomotic techniques. Chirurg. 2011;82(1):7–13. 10.1007/s00104-010-1902-x. 21153387 10.1007/s00104-010-1902-x

[13] Terblanche J, Worthley CS, Spence RA, Krige JE. High or low hepaticojejunostomy for bile duct strictures? Surgery. 1990;108(5):828–34. 2237762

[14] Tani M, Terasawa H, Kawai M, Ina S, Hirono S, Uchiyama K, et al. Improvement of delayed gastric emptying in pylorus-preserving pancreaticoduodenectomy: results of a prospective randomized controlled trial. Ann Surg. 2006;243(3):316–20. 10.1097/01.sla.0000201479.84934.ca. 16495694 10.1097/01.sla.0000201479.84934.ca PMC1448934

[15] Herzog T, Belyaev O, Uhl W, Seelig MH, Chromik AM. Hepaticojejunostomy after pancreatic head resection—technical aspects for reconstruction of small and fragile bile ducts with T-tube drainage. Zentralbl Chir. 2012;137(6):559–64. 10.1055/s-0032-1328008. 23264197 10.1055/s-0032-1328008

[16] Herzog T, Belyaev O, Bakowski P, Chromik AM, Janot M, Suelberg D, et al. The difficult hepaticojejunostomy after pancreatic head resection: reconstruction with a T-tube. Am J Surg. 2013;206(4):578–85. 10.1016/j.amjsurg.2013.01.044. 23906984 10.1016/j.amjsurg.2013.01.044

[17] Riediger C, Müller MW, Michalski CW, Hüser N, Schuster T, Kleeff J, et al. T-tube or no T-tube in the reconstruction of the biliary tract during orthotopic liver transplantation: systematic review and meta-analysis. Liver Transpl. 2010;16(6):705–17. 10.1002/lt.22070. 20517904 10.1002/lt.22070

[18] Ikegami T, Taketomi A, Soejima Y, Yoshizumi T, Shimada M, Maehara Y. Characteristics of biliary reconstruction using a T-tube compared with other methods in left-lobe adult living-donor liver transplantation. J Hepatobiliary Pancreat Surg. 2008;15:346–7. 18535778 10.1007/s00534-007-1259-9

[19] Kobayashi T, Sato Y, Yamamoto S, Oya H, Hara Y, Watanabe T, et al. Long-term follow-up of biliary reconstruction and complications in adult living donor liver transplantation: feasibility of duct-to-duct reconstruction with a T-tube stent. Transpl Proc. 2009;41(1):265–7. 10.1016/j.transproceed.2008.10.039. 10.1016/j.transproceed.2008.10.039 19249531

[20] Martinino A, Pereira JPS, Spoletini G, Treglia G, Agnes S, Giovinazzo F. The use of the T-tube in biliary tract reconstruction during orthotopic liver transplantation: an umbrella review. Transpl Rev (Orlando). 2022;36(4):100711. 10.1016/j.trre.2022.100711. 10.1016/j.trre.2022.100711 35843181

[21] Sun N, Zhang J, Li X, Zhang C, Zhou X, Zhang C. Biliary tract reconstruction with or without T-tube in orthotopic liver transplantation: a systematic review and meta-analysis. Expert Rev Gastroenterol Hepatol. 2015;9(4):529–38. 10.1586/17474124.2015.1002084. 25583036 10.1586/17474124.2015.1002084

[22] Song S, Lu T, Yang W, Gong S, Lei C, Yang J, et al. T-tube or no T-tube for biliary tract reconstruction in orthotopic liver transplantation: an updated systematic review and meta-analysis. Expert Rev Gastroenterol Hepatol. 2021;15(10):1201–13. 10.1080/17474124.2021.1903874. 33720798 10.1080/17474124.2021.1903874

[23] Prawdzik C, Belyaev O, Chromik AM, Uhl W, Herzog T. Surgical revision of hepaticojejunostomy strictures after pancreatectomy. Langenbecks Arch Surg. 2015;400(1):67–75. 10.1007/s00423-014-1246-y. 25277247 10.1007/s00423-014-1246-y

[24] Wang WG, Babu SR, Wang L, Chen Y, Tian BL, He HB. Use of Clavien-Dindo classification in evaluating complications following pancreaticoduodenectomy in 1,056 cases: a retrospective analysis from one single institution. Oncol Lett. 2018;16(2):2023–9. 10.3892/ol.2018.8798. 30008896 10.3892/ol.2018.8798 PMC6036274

[25] Asano T, Natsume S, Senda Y, Sano T, Matsuo K, Kodera Y, Hara K, Ito S, Yamao K, Shimizu Y. Incidence and risk factors for anastomotic stenosis of continuous hepaticojejunostomy after pancreaticoduodenectomy. J Hepatobiliary Pancreat Sci. 2016;23(10):628–35. 10.1002/jhbp.385. 27474880 10.1002/jhbp.385

[26] Bednarsch J, Trauwein C, Neumann UP, Ulmer TF. Komplikationsmanagement nach Gallengangschirurgie. Chirurg. 2020;91(1):29–36. 10.1007/s00104-019-01059-9. 31691143 10.1007/s00104-019-01059-9

[27] Javed AA, Mirza MB, Sham JG, Ali DM, Jones GFT, Sanjeevi S, Burkhart RA, Cameron JL, Weiss MJ, Wolfgang CL, He J. Postoperative biliary anastomotic strictures after pancreaticoduodenectomy. HPB (Oxford). 2021;23(11):1716–21. 10.1016/j.hpb.2021.04.008. 34016543 10.1016/j.hpb.2021.04.008

[28] Herzog T, Belyaev O, Bakowski P, Chromik A, Janot M, Suelberg D, Uhl W, Seelig M. The difficult hepaticojejunostomy after pancreatic head resection: reconstruction with a T tube. Am J Surg. 2013;206(4):578–85. 10.1016/j.amjsurg.2013.01.044. 23906984 10.1016/j.amjsurg.2013.01.044

[29] Vest M, Ciobanu C, Nyabera A, Williams J, Marck M, Landry I, Sumbly V, Iqbal S, Shah D, Nassar M, Nso N, Rizzo V. Biliary anastomosis using T-tube versus no T-tube for liver transplantation in adults: a review of literature. Cureus. 2022;14(4):e24253. 10.7759/cureus.24253. 35602800 10.7759/cureus.24253 PMC9117859

[30] Genest JF, Nanos E, Grundfest-Broniatowski S, Vogt D, Hermann RE. Benign biliary strictures: an analytic review (1970 to 1984). Surgery. 1986;99(4):409–13. 3952666

[31] Himmelfarb R. (2009). Gallengangskomplikationen nach orthotoper Lebertransplantation mit und ohne T-Drainage. Dissertation, Universität Heidelberg. http://d-nb.info/1023708957/34.

[32] Akamatsu N, Sugawara Y, Hashimoto D. Biliary reconstruction, its complications and management of biliary complications after adult liver transplantation: a systematic review of the incidence, risk factors and outcome. Transpl Int. 2011;24(4):379–92. 10.1111/j.1432-2277.2010.01202.x. 21143651 10.1111/j.1432-2277.2010.01202.x

[33] López-Andújar R, Maupoey J, Escrig J, Granero P, Vila JJ, Ibáñez V, Boscá A, García-Eliz M, Benlloch S, Orbis JF, Montalvá EM. Selective indication of T-tube in liver transplantation: prospective validation of the results of a randomized controlled trial. Transplantation Proceedings. 2019;51(1):44–49. 10.1016/j.transproceed.2018.03.133. 10.1016/j.transproceed.2018.03.133 30736977

[34] Strücker S, Neuhaus P, Seehofer D. Prävention und Behandlung von Galleleckagen nach ausgedehnter Leberresektion durch eine neuartige Gallengangsdrainage. German Med Sci GMS Publishing House. 2013;Doc13dgch486. 10.3205/13dgch486.

